# Pooled Multicenter Analysis of Cardiovascular Safety and Population Pharmacokinetic Properties of Piperaquine in African Patients with Uncomplicated Falciparum Malaria

**DOI:** 10.1128/AAC.01848-19

**Published:** 2020-06-23

**Authors:** Thanaporn Wattanakul, Bernhards Ogutu, Abdunoor M. Kabanywanyi, Kwaku-Poku Asante, Abraham Oduro, Alex Adjei, Ali Sie, Esperanca Sevene, Eusebio Macete, Guillaume Compaore, Innocent Valea, Isaac Osei, Markus Winterberg, Margaret Gyapong, Martin Adjuik, Salim Abdulla, Seth Owusu-Agyei, Nicholas J. White, Nicholas P. J. Day, Halidou Tinto, Rita Baiden, Fred Binka, Joel Tarning

**Affiliations:** a Mahidol-Oxford Tropical Medicine Research Unit, Faculty of Tropical Medicine, Mahidol University, Bangkok, Thailand; b Centre for Tropical Medicine and Global Health, Nuffield Department of Medicine, University of Oxford, Oxford, United Kingdom; c INDEPTH Network, Accra, Ghana; d Centre for Clinical Research, Kenya Medical Research Institute, Nairobi, Kenya; e Ifakara Health Institute, Ifakara, Tanzania; f Kintampo Health Research Centre, Kintampo, Ghana; g Navrongo Health Research Centre, Navrongo, Ghana; h Dodowa Health Research Centre, Dodowa, Ghana; i Nouna Health Research Centre, Nouna, Burkina Faso; j Centro de Investigaçãoem Saúde de Manhiça, CISM, Manhiça, Mozambique; k Clinical Research Unit of Nanoro (IRSS-URCN), Nanoro, Burkina Faso; l University for Health and Allied Sciences, Ho, Ghana; m WorldWide Antimalarial Resistance Network, Oxford, United Kingdom

**Keywords:** piperaquine, cardiovascular safety, QT prolongation, population pharmacokinetic-pharmacodynamic model, antimalarial agents, malaria, population pharmacokinetics

## Abstract

Dihydroartemisinin-piperaquine has shown excellent efficacy and tolerability in malaria treatment. However, concerns have been raised of potentially harmful cardiotoxic effects associated with piperaquine. The population pharmacokinetics and cardiac effects of piperaquine were evaluated in 1,000 patients, mostly children enrolled in a multicenter trial from 10 sites in Africa. A linear relationship described the QTc-prolonging effect of piperaquine, estimating a 5.90-ms mean QTc prolongation per 100-ng/ml increase in piperaquine concentration. The effect of piperaquine on absolute QTc interval estimated a mean maximum QTc interval of 456 ms (50% effective concentration of 209 ng/ml). Simulations from the pharmacokinetic-pharmacodynamic models predicted 1.98 to 2.46% risk of having QTc prolongation of >60 ms in all treatment settings. Although piperaquine administration resulted in QTc prolongation, no cardiovascular adverse events were found in these patients. Thus, the use of dihydroartemisinin-piperaquine should not be limited by this concern. (This study has been registered at ClinicalTrials.gov under identifier NCT02199951.)

## Abstract (precis)

Dihydroartemisinin-piperaquine has shown excellent efficacy and tolerability in malaria treatment. However, concerns have been raised of potentially harmful cardiotoxic effects associated with piperaquine. The population pharmacokinetics and cardiac effects of piperaquine were evaluated in 1,000 patients, mostly children enrolled in a multicenter trial from 10 sites in Africa. A linear relationship described the QTc-prolonging effect of piperaquine, estimating a 5.90-ms mean QTc prolongation per 100-ng/ml increase in piperaquine concentration.

## INTRODUCTION

Malaria is a life-threatening disease. The World Health Organization (WHO) reported an estimated 219 million malaria cases and 435,000 malaria-related deaths in 2017. Children aged under 5 years are the most vulnerable group, which accounted for 61% (266,000) of all malaria deaths worldwide (1). Dihydroartemisinin-piperaquine is a WHO-recommended artemisinin-based combination therapy (ACT) that has demonstrated excellent efficacy and tolerability in clinical trials (2–8). This drug is given once daily for 3 days, administered according to a weight-based regimen (8–10). Dihydroartemisinin has a rapid but short-lived parasite killing effect (drug elimination half-life of 1 to 2 h), responsible for substantial parasite killing during the first 3 days of treatment. Piperaquine has a long terminal elimination half-life of 20 to 30 days and is responsible for eliminating residual parasites. The long half-life also protects against malaria reinfections for up to 30 days posttreatment (11–13). Electrocardiographic QT prolongation has been observed in patients and healthy volunteers following piperaquine administration (6, 7, 14, 15). QT prolongation is a risk factor for polymorphic ventricular tachycardia (*torsades de pointes* [TdP]), which may lead to ventricular fibrillation and sudden cardiac death. However, reported QT prolongation after piperaquine administration has not been linked to clinically significant cardiovascular adverse events (16–18). A recent, large, systematic meta-analysis reported that the risk of sudden unexplained death associated with dihydroartemisinin-piperaquine was not higher than the baseline rate of sudden cardiac death in the age-matched population (19). The cardiovascular safety of dihydroartemisinin-piperaquine has been described previously in patients and volunteers. The majority of studies have been in adults. Evaluations and quantifications of the relationship between piperaquine concentration and QT prolongation in children with uncomplicated falciparum malaria in a large-scale multicenter treatment setting are very limited. The main aim of this study was to develop a population pharmacokinetic-pharmacodynamic model to describe and quantify the relationship between piperaquine exposure and QT prolongation to assess cardiovascular safety in patients with uncomplicated malaria (*n* = 1,000) from 10 different sites in Africa.

## RESULTS

### Patient enrollment and demographics.

A total of 11,028 patients with uncomplicated malaria were enrolled in the study (see Fig. S1 in the supplemental material). A total of 10,925 were treated with dihydroartemisinin-piperaquine. Dihydroartemisinin-piperaquine was well tolerated and highly effective in the treatment of uncomplicated malaria. The risk of recurrent symptomatic malaria was low (0.5%), mostly occurring in children younger than 5 years of age (76%) (2). Adverse events were reported in only 5% of patients. The most frequently reported adverse events were graded as mild, including infections and infestations (3.24%), and gastrointestinal disorders (1.37%) (17). A total of 1,305 patients were randomized to the nested cohort study. Thirty patients were lost to follow-up, 11 patients had baseline mean Fridericia-corrected QT intervals (QTc_F_) of >450 ms, and 262 patients had incomplete study procedures (i.e., incomplete electrocardiogram [ECG] measurements and/or incomplete pharmacokinetic samples). Data from 1,000 patients were included for the current pharmacokinetic-pharmacodynamic analysis. Out of 1,000 patients, 299 were from Burkina Faso, 442 were from Ghana, 89 were from Mozambique, and 170 were from Tanzania. Almost 70% of the patients were children aged <12 years. Similar numbers of male and female patients were enrolled (48.2% versus 51.8%, respectively). ECG measurements were recorded in patients in the nested cohort study. Maximum QTc prolongations occurred on day 3 after piperaquine administration (i.e., after the last dose of treatment) in most of the patients. However, no clinical abnormalities as a result of cardiac adverse events were reported in this study. The study diagram is shown in Fig. S1, and the baseline demographics are presented in Table 1.

**TABLE 1:** Baseline patient characteristics

Parameter	Value(s) for:				
	Burkina Faso (*n* = 299)	Ghana (*n* = 442)	Mozambique (*n* = 89)	Tanzania (*n* = 170)	Total (*n* = 1,000)
Patient enrollment					
Age group [*n* (%)]					
<1 yr	5 (0.5)	1 (0.1)	0	0	6 (0.6)
1 to <5 yr	93 (9.3)	116 (11.6)	2 (0.2)	27 (2.7)	238 (23.8)
5 to <12 yr	143 (14.3)	184 (18.4)	49 (4.9)	74 (7.4)	450 (45.0)
12 to <18 yr	18 (1.8)	64 (6.4)	22 (2.2)	23 (2.3)	127 (12.7)
≥18 yr	40 (4.0)	77 (7.7)	16 (1.6)	46 (4.6)	179 (17.9)
Sex [*n* (%)]					
Male	142 (14.2)	203 (20.3)	47 (4.7)	90 (9.0)	482 (48.2)
Female	157 (15.7)	239 (23.9)	42 (4.2)	80 (8.0)	518 (51.8)
Patient characteristics [median (IQR)]					
Age (yr)	6 (4–9)	7.5 (4–13)	11 (9–14)	10 (6–18)	7.5 (5–12)
Body weight (kg)	17 (13–23)	22 (16–42)	31 (26–43)	22 (18–45)	21 (15–38)
Body temperature (°C)	37.4 (36.8–37.7)	37.0 (36.5–38.0)	37.2 (36.5–38.2)	37.7 (37.0–38.9)	37.2 (36.7–38.0)
Pulse rate (beats/min)	100 (87–111)	110 (90–126)	91 (76–111)	99 (84–112)	54 (87–120)
Parasite density (no. of parasites/μl)	960 (440–7,605)	13,466 (1, 210)	23,951 (7,981–74,824)	140 (33–787)	2,537 (297–25,418)
Hemoglobin (g/dl)	10.6 (9.5–11.5)	10.9 (9.8–11.7)	10.8 (9.4–11.9)	10.7 (9.4–11.9)	10.7 (9.6–11.7)
Total bilirubin (μmol/liter)	9.8 (6.6–15.4)	14.0 (9.1–23.6)	17.0 (12.0–25.0)	18.1 (8.3–26.7)	13.4 (8.0–22.9)
ALT (U/liter)	24.3 (18.2–31.1)	20.9 (15.3–31.5)	26.0 (22.0–34.0)	29.1 (21.8–35.6)	24.0 (17.4–33.0)
AST (U/liter)	25.2 (19.1–32.5)	28.4 (22.1–37.9)	33.0 (28.0–44.0)	15.5 (9.6–21.7)	25.9 (18.8–34–9)
BUN (mmol/liter)	2.9 (2.2–3.7)	3.4 (2.4–4.6)	13.0 (11.0–16.0)	6.3 (4.4–10.0)	3.6 (2.6–5.6)
Serum creatinine (μmol/liter)	30.5 (25.0–38.6)	47.8 (38.3–65.3)	44.6 (38.5–53.9)	77.0 (54.0–99.5)	43.9 (32.0–64.0)
Potassium (mmol/liter)	4.2 (4.0–4.4)	3.8 (3.5–4.2)	4.1 (3.9–4.5)	4.4 (3.7–5.7)	4.05 (3.7–4.4)
Chloride (mmol/liter)	102.8 (95.8–105.3)	101.2 (97.0–107.4)	100.0 (99.0–103.0)	108.5 (98.0–114.0)	102.2 (97.0–106.9)

### Population pharmacokinetic modeling of piperaquine.

**FIG 1:**
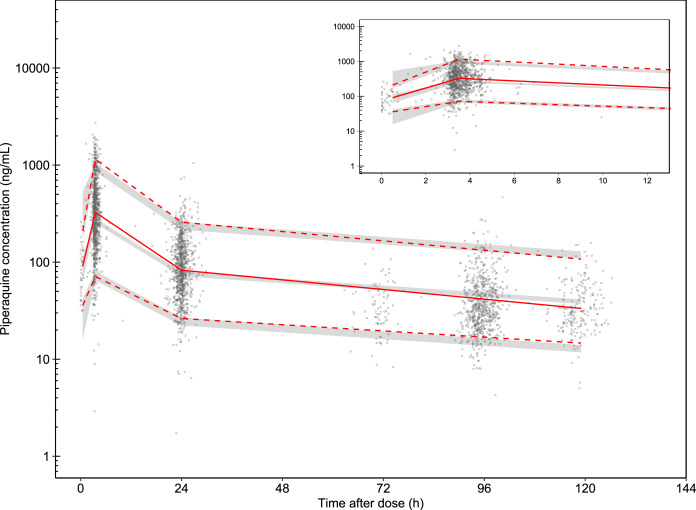
Visual predictive check of the final piperaquine pharmacokinetic model. The open circles represent the observed piperaquine concentrations. Solid red lines represent the 50th percentiles of the observations, and dashed red lines represent the 5th and 95th percentiles of the observations. The shaded areas represent the 95% confidence intervals of each simulated percentile (*n* = 2,000).

A total of 2,989 piperaquine plasma concentrations from 1,000 patients were included in this population pharmacokinetic analysis. A total of 1.44% of observed data were below the limit of quantification and were omitted during model development. The pharmacokinetic sampling of piperaquine was conducted only up to 7 days after the first dose, resulting in limited ability to describe the disposition and elimination phase accurately. Thus, a frequentist prior approach was applied, using information from a pooled pharmacokinetic piperaquine meta-analysis (10). The prior model used was a three-compartment disposition model with two transit absorption compartments, allometric scaling of body weight on clearance and volume parameters, age-related enzyme maturation of elimination clearance, and dose-occasion as a covariate on relative bioavailability. Implementing this prior approach resulted in a stable model with reasonable parameter estimates close to that previously published for piperaquine (Table 2). No additional significant covariate relationships were found for the patients studied here. Goodness of fits of the final model demonstrated an adequate descriptive performance (Fig. S2). Simulation-based diagnostics (visual predictive check; *n* = 2,000 simulations) resulted in satisfactory predictive performance of the final model (Fig. 1). The numerical predictive check (*n* = 2,000) resulted in 2.0% (95% confidence interval [CI], 1.8% to 3.1%) and 3.3% (95% CI, 1.9% to 3.1%) of piperaquine observations below and above the simulated 95% prediction interval, respectively. The individually predicted piperaquine concentration-time profiles from the final pharmacokinetic model were incorporated into the pharmacokinetic-pharmacodynamic model to describe the relationship between piperaquine concentration and QT interval (i.e., sequential modeling approach) (20).

**TABLE 2:** Pharmacokinetic parameters from the final population pharmacokinetic model for piperaquine^*a*^

Parameter	Population estimate^*b*^ (% RSE)^*c*^	95% CI^*c*^	IIV/IOV (% CV)^*b*^ (% RSE)^*c*^	95% CI^*c*^
*F*	1 fixed		38.2 (3.90), 42.8 (2.07)	36.0–42.3, 43.5–47.8
MTT (h)	2.13 (1.11)	2.09–2.18	37.5 (9.83), 44.7 (1.20)	36.1–52.8, 46.2–48.7
CL/*F* (liter/h)	53.1 (2.77)	50.2–56.1		
*V_C_*/*F* (liter)	1,730 (8.04)	1,441–1,991	90.5 (19.8)	31.4–165
*Q*_1_/*F* (liter/h)	282 (5.60)	249–310		
*V_P_*_1_/*F* (liter)	3,290 (5.10)	2,949–3,595	23.4 (24.1)	17.2–39.9
*Q*_2_/*F* (liter/h)	82.9 (2.42)	78.9–86.6	27.1 (11.9)	24.0–36.7
*V_P_*_2_/*F* (liter)	25,100 (1.77)	24,170–25,925	31.8 (1.01)	32.0–33.3
Dose occasion effect on *F*	0.237 fixed			
AGE_50_ (yr)	0.575 fixed			
Hill	5.51 fixed			
σ	0.198 (4.13)	0.167–0.232		

a Abbreviations: F, relative bioavailability; MTT, mean transit time; CL/F, apparent oral clearance; V_C_/F, apparent central volume of distribution; Q/F, intercompartmental clearance; V_P_/F, apparent peripheral volume of distribution; AGE_50_, the age to reach 50% of the full maturation of the elimination clearance; Hill, the shape function in the maturation equation; σ, residual unexplained error of drug measurements (variance); IIV, interindividual variability; IOV, interoccasion variability.

b Computed population mean parameter estimates from NONMEM. Parameter estimates are based on the typical individual in the prior population with a body weight of 54 kg. IIV and IOV were implemented as an exponential function and are presented as the coefficient of variation (%CV), calculated as $100\times \sqrt{\text{exp}\left(\text{estimate}\right)\;-\;1}$.

c Based on nonparametric bootstrap diagnostics (*n* = 1,000). Parameter precision is presented as relative standard deviation (%RSE), calculated as $100\times \frac{\text{standard deviation}}{\text{mean value}}$.

### QT interval correction methods.

The QT interval depends on heart rate. To normalize QT intervals for comparison, a correction is applied to the RR interval. This is usually a power function, such as that described by Bazett (0.5) or Fridericia (0.33). A total of 2,907 pretreatment (day 1) QT and RR interval measurements were used to estimate the optimal study-specific rate correction factor. Ordinary linear regression analysis estimated the correction factor to be 0.476 (95% CI, 0.468 to 0.484). This estimated study-specific correction factor (SSB) then was applied to all QT interval measurements throughout the study (QTc_SSB_). Additionally, separate correction factors were estimated using the QT and RR intervals measured on day 3 and day 7. The estimated correction factors for day 3 and day 7 were 0.442 (95% CI, 0.433 to 0.450) and 0.435 (95% CI, 0.421 to 0.449), respectively. The QT interval measurements then were corrected using the specific correction factor estimated for each day (QTc_DAYS_). The traditionally used Fridericia (QTc_F_) and Bazett (QTc_B_) corrections were also applied to all data for completion. The slope of the linear regression between the corrected QT versus RR intervals was used to evaluate the performance of each correction method (21). A slope close to zero represents a complete correction of the QTc calculations across the heart rate range and a consistent and appropriate performance of the method. The corrected QT interval using a study-specific correction factor (QTc_SSB_) showed the least dependence on the heart rate, with the estimated slope not significantly different from zero (slope, 0.00180; *P* = 0.332). All other correction methods showed various degrees of bias, with regression slopes of 0.00751, −0.0137, and 0.0877, for QTc_DAYS_, QTc_B_, and QTc_F_, respectively (*P* < 0.0001 for all slopes). Linear regressions of QTc intervals and RR intervals of each correction method are presented in Fig. S3.

### Clinical determinants of QTc prolongation.

**FIG 2:**
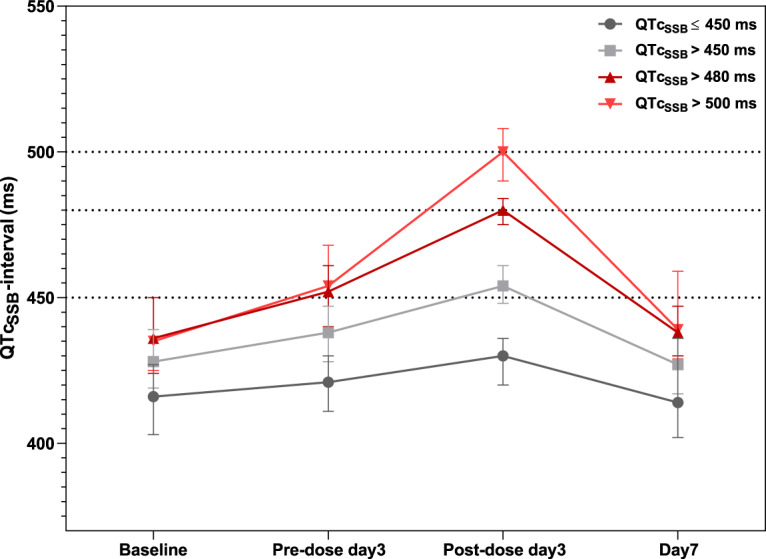
Observed QTc_SSB_ intervals, stratified by ECG measurement schedule. The solid lines and error bars represent the medians and interquartile ranges of QTc_SSB_ intervals recorded at each ECG measurement occasion, stratified by QTc interval threshold categories.

Statistical analyses were performed to evaluate clinical determinants associated with QTc prolongation in each patient stratum of QTc prolongation. Patients were categorized based on the threshold limits suggested in the ICH guideline (22). Both absolute QTc interval and QTc interval prolongation (i.e., increase from baseline; ΔQTc interval) were evaluated. The study-specific correction factor (QTc_SSB_; α = 0.476) was used in this analysis. Overall, patients had the highest QTc_SSB_ intervals on day 3 after the third and last dose of piperaquine. The prolongation was transient and the QTc_SSB_ intervals returned to approximately baseline values on day 7 (Fig. 2).

When allocating patients into different ΔQTc interval strata, 64.2% (638/994), 28.8% (286/994), and 7.0% (70/994) of patients presented a ΔQTc_SSB_ interval of ≤30 ms, 31 to 60 ms, and >60 ms, respectively. The group of patients with ΔQTc_SSB_ intervals of ≤30 ms was used as the reference for these comparisons. Patients with higher ΔQTc_SSB_ intervals had significantly higher body temperature at enrollment (*P* < 0.0001). The baseline QTc_SSB_ interval at enrollment was significantly different in each subgroup (*P* < 0.001), where patients with longer ΔQTc_SSB_ intervals had shorter baseline values. Additionally, the median piperaquine peak concentration (*C*_max_) was significantly higher (*P* < 0.001) in patients with a ΔQTc_SSB_ interval of 31 to 60 ms, but it did not reach significance in patients with QTc_SSB_ intervals of >60 ms (*P* = 0.083), most likely due to a much smaller number of patients in this group. No other clinical determinants were significantly different between groups.

When allocating patients into different absolute QTc interval strata, 48.4% (481/994), 41.0% (408/994), 7.8% (78/994), and 2.7% (27/994) of patients presented a maximum QTc_SSB_ interval of ≤450 ms, 451 to 480 ms, 481 to 500 ms, and >500 ms, respectively. The percentage of female/male patients was similar in all strata (approximately 50%). The baseline QTc_SSB_ interval at enrollment was significantly different in each stratum (*P* < 0.001), where patients with longer QTc_SSB_ intervals also had higher baseline values. The median piperaquine *C*_max_ was significantly different among the groups (*P* < 0.0001), with a gradual increase with increasing QTc_SSB_ interval. No other covariates were significantly different between groups. The results of the statistical analysis of clinical determinants of QTc prolongation are shown in Table 3.

**TABLE 3:** Clinical determinants associated with QTc prolongation

Factor	Clinical determinants associated with:													
	ΔQTc_SSB_ interval prolongation						Absolute QTc_SSB_ interval prolongation							
	≤30 ms^*a*^	31–60 ms	>60 ms	*P* value for:			≤450 ms^*a*^	451–480 ms	481–500 ms	>500 ms	*P* value for:			
				≤30 ms vs 31–60 ms	≤30 ms vs >60 ms	All groups					≤450 ms vs 451–480 ms	≤450 ms vs 481–500 ms	450 ms vs >500 ms	All groups
No. (%) of patients	638 (64.2)	286 (28.8)	70 (7.0)				481 (48.4)	408 (41.0)	78 (7.8)	27 (2.7)				
No. (%) female	322 (50.5)	161 (56.3)	33 (47.1)	0.117	0.617	0.186	229 (47.6)	234 (57.4)	39 (50.0)	14 (50.0)	0.004^*b*^	0.715	0.696	0.036^*b*^
No. (%) with hypokalemia (<3.5 mmol/liter)	87 (13.6)	52 (18.2)	7 (10.0)	0.090	0.463	0.101	65 (13.5)	63 (15.4)	11 (14.1)	6 (22.2)	0.444	0.860	0.247	0.580
Body temp (°C) [median (IQR)]	37.1 (36.6–37.8)	37.5 (36.8–38.4)	37.6 (36.8–38.6)	<0.0001^*b*^	0.006^*b*^	<0.0001^*b*^	37.2 (36.6–38.0)	37.3 (36.7–38.0)	37.4 (36.8–37.9)	37.0 (36.5–37.3)	>0.999	>0.999	0.543	0.437
Age (yr) [median (IQR)]	7 (5–14)	8 (5–12)	8 (5–10)	>0.999	>0.999	0.873	8 (5–15)	7 (4–13)	8 (5–11)	9 (6–12)	0.675	>0.999	>0.999	0.489
Potassium (mmol/liter) [median (IQR)]	4.05 (3.70–4.47)	4.01 (3.70–4.40)	4.16 (3.80–4.49)	0.332	0.383	0.111	4.10 (3.70–4.50)	4.00 (3.70–4.40)	4.01 (3.60–4.38)	3.88 (3.60–4.30)	0.351	0.837	0.164	0.126
Baseline QTc_SSB_ interval (ms) [median (IQR)]	425 (413–435)	422 (406–434)	415 (391–433)	0.015^*b*^	0.002^*b*^	0.0004^*b*^	416 (403–427)	428 (419–439)	436 (424–450)	435 (425–450)	<0.0001^*b*^	<0.0001^*b*^	<0.0001^*b*^	<0.0001^*b*^
Piperaquine *C*_max_ (ng/ml) [median (IQR)]	694 (442–1,030)	814 (546–1,110)	830 (550–1,120)	0.0004^*b*^	0.083	0.0003^*b*^	651 (421–960)	783 (517–1,100)	962 (656–1,320)	905 (664–1,150)	<0.0001^*b*^	<0.0001^*b*^	0.045^*b*^	<0.0001^*b*^

a Reference groups.

b Statistically significant.

The possible drug-drug interactions of piperaquine and concomitant medications that prolong the QTc interval were also investigated. Twelve patients received at least one medication, listed on www.Crediblemeds.org (accessed 19 June 2019) as drugs that prolong the QTc interval, during the study period. The concomitant medications were metronidazole, ketoconazole, fluconazole, ciprofloxacin, furosemide, and metoclopramide. Among these twelve patients, five patients had a ΔQTc_SSB_ interval of ≤30 ms, six patients had a ΔQTc_SSB_ interval of 31 to 60 ms, and one patient had a ΔQTc_SSB_ interval of >60 ms. With respect to absolute QTc interval, seven patients had a QTc_SSB_ interval of ≤450 ms, four patients had a QTc_SSB_ interval of 451 to 480 ms, and one patient had a QTc_SSB_ interval of 481 to 500 ms.

### Relationship between piperaquine concentration and QTc interval.

**FIG 3:**
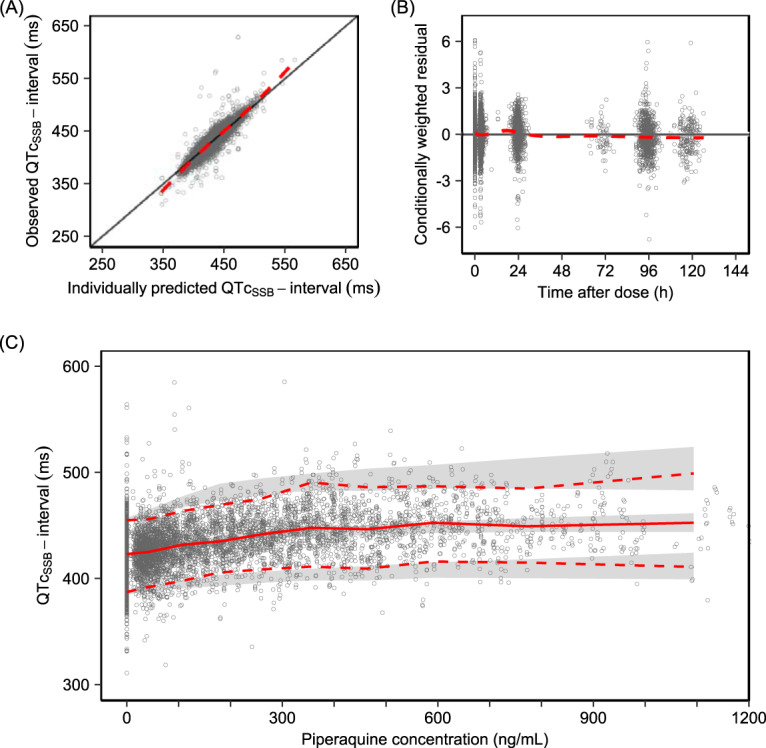
Diagnostics of the final pharmacokinetic-pharmacodynamic model. (A and B) Goodness-of-fit plots showing observed QTc_SSB_ interval versus individually predicted QTc_SSB_ interval (A) and conditionally weighted residual versus time after dose (B). The solid black lines represent the line of identity, and the dashed red lines represent a local polynomial regression fitting of all observations (i.e., trend line). (C) Visual predictive check of the model describing the relationship between piperaquine concentrations and absolute QTc_SSB_ intervals using an *E*_max_ function (*n* = 2,000). The open circles represent the observations. The solid red line represents the 50th percentile of the observations, and dashed red lines represent the 5th and 95th percentiles of the observations. The shaded areas represent the 95% confidence intervals of each simulated percentile.

To quantify the magnitude of absolute QTc interval prolongation resulting from piperaquine administration, a population pharmacokinetic-pharmacodynamic analysis was performed. The absolute QT intervals were corrected for heart rate using the study-specific correction factor (QTc_SSB_) and evaluated with nonlinear mixed-effects modeling. There was a 4.87-ms QTc_SSB_ prolongation per 100-ng/ml increase in piperaquine plasma concentration when described by a linear concentration-response relationship. This model was improved further by implementing an *E*_max_ function (ΔOFV = −1,886). A stepwise covariate search demonstrated that potassium concentration had a significant impact on the estimated QTc_SSB_ interval at baseline (QTc_Baseline_) and that age influenced the 50% effective concentration (EC_50_) significantly. This covariate effect estimated that a 1-mmol/liter increase in potassium level resulted in a 1.06-ms (0.25%) decrease in QTc_Baseline_, but the variation in potassium concentrations over the study period was relatively narrow (interquartile range [IQR], 3.70 to 4.48). Thus, the effect of potassium level on the QTc_Baseline_ was considered to have negligible clinical relevance and was not included as a covariate in the final model. Age as an effect on EC_50_ was the only covariate that was retained in the final model (ΔOFV = −53.3). The parameter estimates from the final pharmacokinetic-pharmacodynamic model describing the effect of piperaquine on the absolute QTc_SSB_ intervals are summarized in Table 4. Goodness of fits and visual predictive checks of the final model are shown in Fig. 3.

**TABLE 4:** Parameter estimates from the final pharmacokinetic-pharmacodynamic model for the piperaquine effect on absolute QTc_SSB_ interval^*a*^

Parameter	Population estimate^*b*^ (% RSE)^*c*^	95% CI^*c*^	IIV %CV (% RSE)^*c*^	95% CI^*c*^
QTc_Baseline_ (ms)	421 (0.15)	420–423	17.0^*d*^ (3.12)	16.0–18.0
*E*_max_ (ms)	35 (11.0)	29.0–44.2	49.1^*e*^ (13.0)	34.1–62.4
EC_50_ (ng/ml)	209 (16.7)	155–296	119.3^*e*^ (9.28)	88.7–152
γ	1.69 (11.6)	1.36–2.17		
Effect of age on EC_50_ (%)	4.10 (19.5)	2.68–5.88		
σ (ms)	11.6 (5.74)	10.5–13.2		

a Abbreviations: QTc_Baseline_, baseline value of the QTc_SSB_ interval at enrollment; *E*_max_, maximum QTc_SSB_ interval associated with drug effect; EC_50_, piperaquine concentration needed to achieve 50% of the maximum drug effect; γ, shape function of the *E*_max_ model; σ, additive residual error (variance) of QTc_SSB_ interval measurements; IIV, interindividual variability.

b Computed population mean parameter estimates from NONMEM.

c Based on nonparametric bootstrap diagnostics (*n* = 1,000). Parameter precision is presented as relative standard deviation (%RSE), calculated as $100\times \frac{\text{standard deviation}}{\text{mean value}}$.

d Additive interindividual variability, presented as absolute variability on an arithmetic scale.

e Exponential interindividual variability, presented as the coefficient of variation (%CV), calculated as $100\times \sqrt{\text{exp}\left(\text{estimate}\right)-1}$.

Separate analyses describing the relationship of piperaquine concentration and ΔQTc interval were conducted using four different correction methods (QTc_F_, QTc_B_, QTc_SSB_, and QTc_DAYS_). A linear relationship of piperaquine concentrations versus ΔQTc_F_, ΔQTc_B_, ΔQTc_SSB_, and ΔQTc_DAYS_ estimated a QTc prolongation of 7.97 ms, 5.30 ms, 5.90 ms, and 4.11 ms, respectively, per 100-ng/ml increase in piperaquine concentration. Further details of these analyses are provided in the supplemental material.

### Population-based simulations of clinical scenarios.

**FIG 4:**
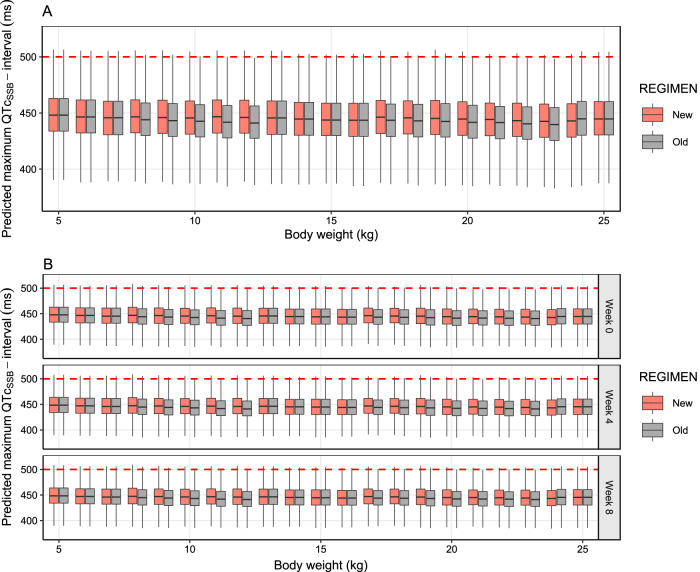
Predicted maximum QTc_SSB_ intervals after different dosing regimens, simulated from the final pharmacokinetic-pharmacodynamic model. The box plots represent the simulated maximum QTc_SSB_ interval, stratified by body weight, in children weighing 5 to 25 kg (data on adults are presented in Fig. S5 in the supplemental material) after receiving the old and new dosing regimen for acute malaria treatment (3-day regimen) (A) and mass drug administration (monthly 3-day regimen) (B). The dashed red lines represent an absolute QTc interval regulatory safety cutoff of 500 ms.

**FIG 5:**
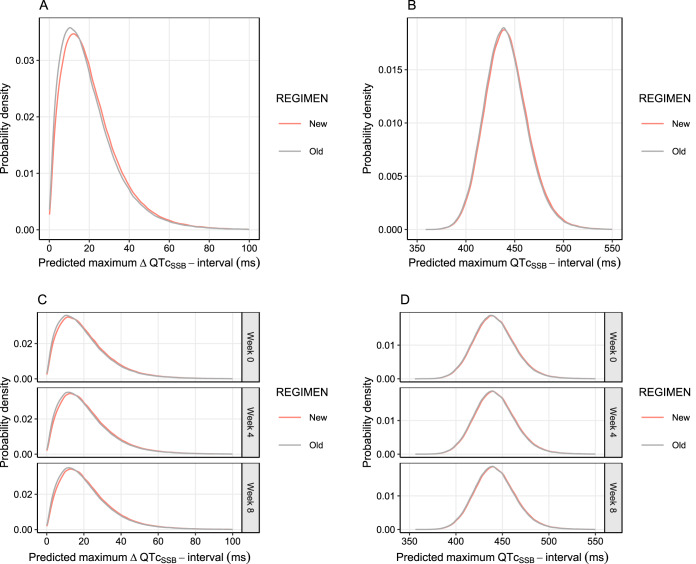
Probability density of maximum QTc_SSB_ intervals and ΔQTc_SSB_ intervals after different dosing regimens, simulated from the final pharmacokinetic-pharmacodynamic model. The graphs shows the probability density distribution of maximum QTc_SSB_ intervals and maximum ΔQTc_SSB_ intervals based on a total of 480,000 simulated patients after receiving the old (gray solid lines) and new (red solid lines) dosing regimens for acute malaria treatment (3-day regimen) (A and B) and for mass drug administration (monthly 3-day regimen) (C and D).

The final pharmacokinetic-pharmacodynamic model describing the effects of piperaquine on the absolute QTc_SSB_ interval was used for large-scale Monte Carlo population simulations of possible clinical scenarios. The simulations included two settings, acute treatment of symptomatic malaria (full 3-day treatment course) and mass drug administration (full 3-day treatment course given once a month for a total of 3 months). Two different dosing recommendations for dihydroartemisinin-piperaquine were evaluated, the old recommendation (2nd edition) and new recommendation (3rd edition) of the WHO guidelines for the treatment of malaria (Table 5). Overall, the simulated maximum QTc_SSB_ interval (QTc_max_) and maximum QTc_SSB_ prolongation (ΔQTc_max_) when using the old and new recommended doses demonstrated similar results when used in both acute treatment and mass drug administration scenarios. For acute malaria treatment, the median predicted QTc_max_ values were 440 ms (95% CI, 401 to 489 ms) and 441 ms (95% CI, 401 to 490 ms) for the old and new piperaquine dosing regimens, respectively. In a mass drug administration setting, the median predicted QTc_max_ values were 440 ms (95% CI, 401 to 490 ms) and 441 ms (95% CI, 402 to 491ms) for the old and new piperaquine dosing regimens, respectively. The simulated total probability of having a QTc_SSB_ interval above 500 ms was 1.1% (11 in 1,000 patients) and 1.2% (12 in 1,000 patients) in acute treatment of malaria using the old and new piperaquine dosing regimen, respectively. Similarly, the simulated total probability of having a QTc_SSB_ interval above 500 ms was 1.2% (12 in 1,000 patients) and 1.3% (13 in 1,000 patients) in mass drug administration settings using the old and new piperaquine dosing regimens, respectively. The probability of having a QTc_max_ of >500 ms, stratified by body weight, is shown in Fig. S5. The simulations showed that predicted ΔQTc_max_ values of more than 60 ms were infrequent (1.98 to 2.25%). Acute treatment resulted in a predicted median ΔQTc_max_ of 16.8 ms (95% CI, 2.31 to 56.9 ms) and 18.0 ms (95% CI, 2.67 to 58.6 ms) for the old and new piperaquine dosing regimens, respectively. Similarly, the median predicted ΔQTc_max_ was 17.6 ms (95% CI, 2.58 to 57.9 ms) and 18.5 ms (95% CI, 2.90 to 59.3 ms) for the old and new piperaquine dosing regimens given in a mass drug administration scenario. Simulated QTc_max_ stratified by body weight are shown in Fig. 4. The distribution of predicted QTc_max_ and ΔQTc_max_ of each clinical scenario is shown in Fig. 5. The probability of having QTc_max_ and ΔQTc_max_ at different threshold levels is shown in Table S3.

**TABLE 5:** Old and new dihydroartemisinin-piperaquine dosing regimen recommended by WHO for the treatment of uncomplicated malaria

Old piperaquine dosing regimen		New piperaquine dosing regimen	
Body wt (kg)	DHA/PQP dose (mg)	Body wt (kg)	DHA/PQP dose (mg)
5–12	20/160	5 to <8	20/160
13–23	40/320	8 to <11	30/240
24–35	80/640	11 to <17	40/320
36–74	120/960	17 to <25	60/480
>74	160/1,280	25 to <36	80/640
		36 to <60	120/960
		60 to <80	160/1,280
		>80	200/1,600

## DISCUSSION

Dihydroartemisinin-piperaquine was well tolerated and highly effective in the treatment of uncomplicated malaria, as reported in this and earlier studies (2, 17, 18). This analysis focused on electrocardiographic QT prolongation, a risk factor for ventricular tachyarrhythmias (TdP). Piperaquine, like several other aminoquinolines and structurally related antimalarial drugs, prolongs the QT interval. Halofantrine is an antimalarial drug that caused marked QT prolongation and was associated with sudden death. It has now been discontinued, but concern over a “class effect” remains. This study sought to characterize the relationship between piperaquine plasma concentrations and QT prolongation and, thereby, gauge the potential risk of dangerous tachyarrhythmias. No clinical abnormalities as a result of cardiac adverse events were reported in this postlicensing study. The individual QTc prolongations recorded in the nested pharmacokinetic and electrocardiographic cohort were not associated with any clinical abnormalities, and 89% of all patients returned to within 20 ms of their baseline values on day 7.

### Population pharmacokinetic modeling of piperaquine.

The use of a frequentist prior model described the population pharmacokinetic properties of piperaquine adequately. Indeed, the application of this technique stabilized the estimation of pharmacokinetic parameters, since the study data alone provided insufficient information to generate plausible parameter estimates. Thus, the final pharmacokinetic parameter estimates in this study were generally in agreement with those reported in the prior model (10). The prior model was developed using pooled individual patient data from 11 clinical studies (8,776 samples from 728 individuals), including participants aged 0.56 to 55 years (68% were children aged <12 years) with uncomplicated malaria (93%) and healthy volunteers (6.9%) from Southeast Asia (35%) and Africa (65%). These characteristics of the meta-analysis study were considered similar to those of the current study population, in that patients were African and 69% of the patients were aged <12 years. The inclusion of a wide range of different patients enabled the identification and quantification of several biologically important covariates, including the impact of body weight and age on the elimination of piperaquine. The inclusion of these covariates when fitting the prior model to the data collected in this study provided results that are more generalizable and, therefore, of higher confidence when using the developed model for simulations. The predicted piperaquine concentrations from the final model were in agreement with the observations, demonstrating that the model was adequate for further pharmacokinetic-pharmacodynamic analysis and translational simulations.

### QT interval correction methods.

Several different rate correction approaches of the QT interval have been proposed with no clear consensus. Among the four different correction methods evaluated, unsurprisingly the correction based on the study-specific data performed better than the two most commonly used correction methods (Bazett and Fridericia). The correction factor estimated on pretreatment data alone (QTc_SSB_) showed an overall better performance than using specific corrections calculated at each day of follow-up (QTc_DAYS_). The reason for this might be an overfitting of the data when estimating correction factors for each day separately. However, when estimating separate correction factors on days 1, 3, and 7, the estimated correction factor decreased gradually when people were recovering from malaria (α = 0.476, 0.442, and 0.435). Thus, this might explain why the Bazett correction (α = 0.5) shows an advantage against the more widely used Fridericia correction (α = 0.33) in most patient studies, whereas the opposite is true in healthy volunteer studies. The study-specific correction factor showed no residual trend when evaluating the relationship between corrected QTc intervals and RR intervals, suggesting this is the most appropriate correction factor in the study population. The estimated correction factor of 0.476 (95% CI, 0.468 to 0.484) was higher than the estimated correction factor of 0.4, previously reported in Karen patients with uncomplicated malaria on the Thailand-Myanmar border (23). A study in infants and young children (aged 1 month to 5 years) with sensorineural hearing loss who underwent ECG screening for congenital long QT syndrome demonstrated that the Bazett correction (α = 0.5) was the most appropriate correction among the commonly used methods (21). However, the computed slope of the regression between QTc and RR intervals, using various correction factors (α) from 0.3 to 0.6, demonstrated that the correction factor α = 0.48 generated a slope equal to zero. This value is very similar to the correction factor estimated in the current study (α = 0.476), including mostly children under the age of 5. The estimated correction presented here was based on a large number of patients from 10 different sites at different ages and body weights and, therefore, should be representative of typical malaria patients in Africa. Estimation of individual correction factors, using linear mixed-effect modeling, was also attempted using pretreatment data. However, only one triplicate ECG measurement during the pretreatment period was available for each patient, which was insufficient to estimate an individual correction factor precisely. Study-specific correction factors or an individual correction factor should be used when possible and if data allow these to be estimated reliably. However, the Bazett correction can be used when data or study design do not allow for these study-specific or individual correction factors to be estimated.

### Clinical determinants of QTc prolongation.

The statistical analysis performed in the current study evaluated and identified biological factors that might influence the QTc interval, especially in the subpopulations presenting with greater QTc prolongation. The analysis revealed that patients who had a ΔQTc_SSB_ interval of >60 ms also had a significantly shorter baseline QTc_SSB_ interval than the other two groups. They also had the highest median body temperature at enrollment (37.6°C; IQR, 36.8 to 38.6°C). Fever has been identified as a factor associated with QTc prolongation in patients with congenital long QT syndrome (24, 25). However, in the general population, fever has been reported as a factor associated with QTc shortening (24, 26, 27). This might partly explain the shorter baseline QTc_SSB_ interval in this group of patients and, therefore, the apparent large QTc_SSB_ interval prolongations as patients recover from malaria. The highest QTc_SSB_ prolongation in most of the patients was observed on day 3 after the last dose of piperaquine administration. This occurred approximately at the same time as fever clearance and might, therefore, reflect an additional QTc prolongation on top of the drug effect. Previous studies report a mean QTc prolongation of 11 to 18 ms (comparing baseline and day 3 values with various heart rate correction methods) in patients receiving antimalarial treatments unlikely to increase the QTc interval (i.e., mefloquine and sulfadoxine-pyrimethamine). The QTc prolongation reported in these studies was explained preliminarily by the resolution of fever associated with the recovery from malaria (23, 28, 29).

The analysis of the absolute QTc interval found a total of 27 (2.7%) patients with an observed maximum QTc_SSB_ interval of >500 ms, and 19 patients (70.4%) in this group also had a ΔQTc_SSB_ interval of >60 ms. These patients also had longer median baseline QTc_SSB_ intervals (435 ms; IQR, 425 ms to 450 ms) than other groups. The longer baseline QTc_SSB_ interval might have resulted in a longer absolute QTc_SSB_ interval at a given piperaquine concentration than that of patients with lower baseline values. Furthermore, the median piperaquine *C*_max_ was significantly higher in patients with maximum QTc_SSB_ intervals of >500 ms than in those with QTc_SSB_ intervals of ≤450 ms but was not different from those of patients with QTc_SSB_ intervals of 451 to 480 ms. Thus, piperaquine concentrations alone did not put patients at high risk of QTc_SSB_ intervals of >500 ms. This could be partially explained by the nonlinear relationship between piperaquine concentration and QTc prolongation. Other factors associated with QTc prolongation were not significantly different between the groups described here. One patient received ciprofloxacin for treatment of dysentery, started on the same day piperaquine was given (no specific stop date recorded). This patient had a maximum ΔQTc_SSB_ interval of 72 ms and a maximum absolute QTc_SSB_ interval of 482 ms on day 7. Although no clinical cardiovascular events occurred in this patient, to minimize the risk of cardiovascular events, the use of medications known to prolong the QTc interval should be avoided or used only when the benefits outweigh the risks during piperaquine administration.

### Relationship between piperaquine concentration and QTc interval.

The final pharmacokinetic-pharmacodynamic model of the absolute QTc interval described the data adequately. The estimated QTc_SSB_ interval at enrollment in the malaria patients in this study was 421 ms, with an interindividual variability of ±17.0 ms. This is close to the upper end compared to healthy adults, who commonly have QTc_F_ intervals in the range of 400 to 423 ms (30). Several factors are known to affect the QT interval, e.g., physical activity, food consumption, genetic factors, age, sex, heart rate, stress, circadian rhythm, and electrolyte imbalances (31, 32). Among these factors, heart rate and circadian rhythm are known to have a large impact and should be taken into consideration when evaluating the effect of drugs on the QT interval. The correction for heart rate was performed to account for this potential bias. The effect of circadian rhythm was evaluated using a cosine function. However, this did not improve the model fit significantly. This is most likely a consequence of ECG measurements being collected only from 8 a.m. to 8 p.m. in this study. Thus, it was not possible to estimate precisely the fluctuation of the QT interval through the whole 24-h period. Maximum daily fluctuations of the QT interval have been reported to be 6.75 to 7.80 ms in healthy adult subjects (31, 33). However, this value could not be incorporated *a priori* in the model, since the circadian pattern in children with malaria has not been well characterized. A thorough QT study, with measurements over a 24-h period, in malaria patients receiving antimalarial drugs that do not have an effect on the QT interval would further our understanding of the circadian rhythm in malaria patients and could benefit cardiotoxicity evaluations of antimalarial drugs in future clinical trials. The *E*_max_ relationship of piperaquine exposure and QTc interval was superior to a linear function in this study, which might be due to a large number of available samples and a wider range of observed piperaquine concentrations, demonstrating a plateau in the QTc response at high piperaquine concentrations. Age was a significant covariate on EC_50_, resulting in lower EC_50_ values in young children than in adults, suggesting a relatively greater QT prolongation in young children than adults at equivalent piperaquine concentrations. This should be taken into consideration when evaluating the safety of piperaquine in young children.

From the separate analysis of ΔQTc interval (see the supplemental material), the estimated QTc_SSB_ prolongation of 5.90 ms per 100-ng/ml increase in piperaquine concentration was similar to that reported previously in Cambodian malaria patients (5.00-ms QTc_F_ prolongation) and healthy Thai volunteers (4.17-ms QTc prolongation, using an individual correction method) receiving dihydroartemisinin-piperaquine (34, 35). A study performed in healthy volunteers who received artefenomel (OZ439) in combination with piperaquine reported a similar effect of piperaquine of 4.75-ms QTc_F_ prolongation per 100-ng/ml increase in piperaquine concentration (36).

### Population-based simulations of clinical scenarios.

Both acute malaria treatment and mass drug administration showed similar patterns of predicted QTc_SSB_ prolongation for the previously recommended WHO treatment and the new increased piperaquine dosage in young children. The simulations suggested that the proportions of piperaquine-related maximum QTc_SSB_ prolongation of more than 60 ms in all settings were relatively small. However, the main concern for potentially dangerous cardiotoxicity is those patients or subjects with the highest drug levels and the greatest QTc prolongation (i.e., >500 ms). The simulated total probability of having a QTc_SSB_ interval above 500 ms was <2% in all simulated scenarios. No potential risk of piperaquine accumulation was identified that may cause a higher risk of QTc_SSB_ prolongation. These results were in agreement with a study in healthy volunteers receiving a standard 3-day dose of dihydroartemisinin-piperaquine for three consecutive months, which demonstrated that the average increases in QTc_F_ intervals were comparable between the first month and third month of dosing, and there was no evidence of cumulative cardiotoxicity reported (37).

Although it is clear that piperaquine increases the QT intervals when used in acute malaria treatment and mass drug administration efforts, these QTc prolongations have not resulted in severe cardiac events and sudden death (17, 18). The results from *in vitro* and animal studies suggested that although piperaquine affects the human ether-à-go-go-related gene (hERG) potassium channel, it demonstrated a low potential to induce TdP, either alone or in combination with dihydroartemisinin (38). In addition, a recent pooled meta-analysis of nearly 200,000 exposed individuals demonstrated that dihydroartemisinin-piperaquine was associated with a low risk of sudden unexplained death, which was not higher than the baseline rate of sudden cardiac death (19).

### Limitations.

There were some limitations associated with this study. An accurate and unbiased evaluation of drug-induced QT prolongation can be problematic, especially in patients with malaria, since the disease itself affects the electrocardiogram and heart rate. The highest QTc prolongation observed on day 3 after the last dose of piperaquine coincides with the recovery of malaria. Moreover, the patients enrolled here had no known predisposing factors for arrhythmias or cardiac conditions. Patients with family histories of sudden death, patients taking drugs that prolong the QT interval, and patients who had a baseline QTc interval of >450 ms were excluded from the study. Thus, results of this analysis may not be representative of patients who are predisposed to having cardiovascular risks and events.

In the current study, dihydroartemisinin-piperaquine was given to fasted patients, and food effects could not be evaluated. A recent study demonstrated that concomitant food, especially high-fat and high-calorie food, increased piperaquine exposure, resulting in an increased QTc prolongation, and suggested that dihydroartemisinin-piperaquine should be given in the fasting state (39). Although high-fat meals should be avoided, normal meals do not substantially alter the absorption of piperaquine (8). The simulations in the mass drug administration setting were extrapolated from acute malaria treatment in the current study. Several clinical factors could be different between the populations receiving mass drug administration and acute malaria patients. The QTc prolongation may be more pronounced in acute malaria treatment than a healthy population or individuals with asymptomatic infections. However, we have shown that there was no accumulation in the risk of QTc prolongation associated with repeated piperaquine administration for prophylactic or elimination treatment.

In conclusion, no clinical abnormalities related to cardiovascular adverse events were reported in the 1,000 patients receiving dihydroartemisinin-piperaquine in this study. The developed population pharmacokinetic-pharmacodynamic model described the relationship between QTc prolongation and piperaquine concentrations, resulting in increasing QTc intervals with increasing concentrations. Simulations from the developed model suggested that the risk of QTc prolongation was similar in the previously and newly recommended dose regimen of dihydroartemisinin-piperaquine, and there was no increase in the risk of cardiotoxicity associated with mass drug administration over several months. Although piperaquine increases the QTc interval, clinical studies have demonstrated that piperaquine had a low potential to induce TdP and a low risk of sudden unexplained death. Therefore, concerns about cardiotoxicity should not limit the current clinical use of dihydroartemisinin-piperaquine. However, screening for cardiac conditions and factors associated with QTc prolongation is recommended to diminish the risk of undesirable adverse events.

## MATERIALS AND METHODS

### Study design and patient enrollment.

This was a postlicensing pharmacovigilance study, evaluating the safety of dihydroartemisinin-piperaquine (Eurartesim) conducted in patients with uncomplicated malaria from four African countries, including Ghana, Tanzania, Burkina Faso, and Mozambique. The study was designed as a prospective, observational, open-label, noncomparative, multicenter study. The protocol was approved by the institutional and national ethics committees in all four countries before patients were enrolled in the study. The study was registered at Clinicaltrials.gov on 1 May 2013 (NCT02199951). Written informed consent was obtained from all patients or from their parents or guardians if they were aged <18 years. Patients were enrolled into two groups, the main study and the nested cohort study with identical inclusion and exclusion criteria. In the main study, it was planned to collect data from 10,000 patients who received dihydroartemisinin-piperaquine treatment to assess the treatment outcome and adverse events. In the nested cohort, the collection of data was planned in 1,000 patients to assess the effect of dihydroartemisinin-piperaquine on electrocardiography. The study flow chart can be seen in Fig. S1 in the supplemental material. Patients with confirmed uncomplicated malaria infection were recruited from the outpatient departments of the public health centers in each study site. The inclusion criteria were (i) age ≥6 months, (ii) weight of ≥5 kg, (iii) ability to tolerate oral medication, and (iv) willingness to participate in the study based on written informed consent. The exclusion criteria were (i) allergy to artemisinin or piperaquine, (ii) history of taking dihydroartemisinin-piperaquine in the previous 4 weeks, (iii) pregnant women, (iv) lactating women, (v) severe malaria, (vi) history of taking medicinal products that are known to prolong the QT interval (i.e., antiarrhythmic, neuroleptic, and certain antimicrobial agents), (vii) family history of sudden unexplained death, and (viii) personal or family history of predisposing cardiac conditions for arrhythmia/QT prolongation (including congenital long-QT syndrome, arrhythmia, and any known QTc_F_ or QTc_B_ of more than 450 ms).

### Laboratory and ECG measurements.

Detailed laboratory assessments were performed at each visit, including white blood cell and red blood cell counts, hemoglobin level, platelet count, total bilirubin, alanine aminotransferase (ALT), aspartate aminotransferase (AST), creatinine, blood urea nitrogen (BUN), serum potassium, and serum chloride. Parasite count was performed under microscopy using thick blood smears. The parasite density was calculated based on 200 white cells counted, assuming 8,000 white cells/μl. Blood samples for pharmacokinetic analysis were collected randomly for each patient at approximately 0, 48, 52, 120, 144, and 168 h after the first dose of dihydroartemisinin-piperaquine administration (1 to 5 samples/patient). ECG measurements were performed using the 12-lead digitalized ELI 150 cardiograph provided by CardiaBase (40). ECG measurements were collected in all patients predose on day 1 (baseline), predose on day 3, postdose on day 3, and on day 7. All measurements were recorded once per occasion, except on day 1 (baseline) and postdose on day 3, which were recorded in triplicate with 1- to 2-min intervals between each reading. The positioning of the 12 leads was standardized for all ECG measurements. The ECGs were recorded at least 3 h apart from food intake and were taken from patients in a relaxed supine position in a quiet room. The ECGs were read by trained and ECG-certified study clinicians. The ECG readers were blinded for the time and the day of the ECG recording. A computer-assisted, semiautomatic, on-screen measurement of the digital ECG waveform was used for the reading (ECG Manager). The ECG reading of each particular patient was performed by the same cardiologist. The QTc_F_ was calculated automatically, and the QT interval was verified by the study clinician. Participants with an average QTc_F_ of  ≥450 ms were excluded from the study and prescribed alternative antimalarial medicines. The complete details of the ECG measurement method have been described in previous studies (17, 18).

### Drug administration.

Dihydroartemisinin-piperaquine was administered based on body weight as a once-daily dosing for 3 days (Table 5). Pediatric (20/160 mg) and adult (40/320 mg) fixed-dose formulations of dihydroartemisinin/piperaquine were used in children and adults, respectively. Dihydroartemisinin-piperaquine administration was directly observed by the research team for all 3 days of dosing in all patients. The drug was given with water, and patients were encouraged to avoid food intake for 3 h before and after dosing. In small children, the tablets were crushed and given on a spoon with water. The dissolution profiles for the crushed and whole tablets of dihydroartemisinin-piperaquine were superimposed, indicating that the absorption should be the same in children taking either a crushed or a whole tablet (41). A full dose was readministered in patients who vomited within 30 min after drug administration. For patients vomiting between 30 and 60 min after drug administration, a half dose was readministered. Redosing was performed once, and rescue treatment was given for unsuccessful redosing.

### Piperaquine quantification.

Piperaquine plasma samples were shipped on dry ice to the Department of Clinical Pharmacology, Mahidol-Oxford Tropical Medicine Research Unit, Bangkok, Thailand, for drug quantification and population pharmacokinetic-pharmacodynamic analyses. Piperaquine plasma concentrations were determined using solid-phase extraction followed by liquid chromatography coupled with tandem mass spectrometry per published methods (42). The lower limit of quantification (LLOQ) was set at 1.50 ng/ml. Three replicates of quality control samples at low, middle, and high concentrations (4.50, 20.0, and 400 ng/ml) were analyzed within each batch of clinical samples to ensure the accuracy and precision of the drug assay. The relative standard deviations (percent coefficient of variation [%CV]) were less than 6% for all quality control samples.

### Population pharmacokinetic modeling of piperaquine.

The population pharmacokinetic analysis was performed using nonlinear mixed-effects modeling in NONMEM software, version 7.3 (Icon Development Solution, Ellicott City, MD). The first-order conditional estimation method with interaction (FOCE-I) was used throughout the model development. Piperaquine has a long terminal elimination half-life of approximately 18 to 28 days; however, the plasma samples were collected only for up to 7 days. A frequentist prior approach (43) was implemented to stabilize the estimation and avoid structural model misspecification due to this limited sampling design. Regarding this, the $PRIOR record was implemented to allow a Bayesian penalty function to be added to the NONMEM objective function. This constrains the parameter estimates (i.e., fixed and random effects) in the model, stabilizing the estimates obtained from insufficient and/or uninformative data. The prior pharmacokinetic parameters of piperaquine were adopted from an individual participant data meta-analysis (8,776 samples from 728 individuals), which included children aged <12 years (68%) and adults (32%) with uncomplicated malaria (93%) and healthy volunteers (6.9%) from Southeast Asia (35%) and Africa (65%) (10). The prior model consisted of a three-compartment disposition model with two transit absorption compartments. The identified and quantified covariates were body weight (fixed allometric function), enzyme maturation (maturation half-time of 7 months), and dose occasion (24% increased relative bioavailability between each consecutive dose of piperaquine). The model structure, parameter estimates, and covariate effects of this model were applied using a frequentist prior methodology as described above. Pharmacokinetic parameters were assumed to be log-normally distributed, and the interindividual variability was implemented as an exponential function (equation 1).

(1) $$\theta_{i}=\theta \times e^{\eta_{i,\theta }}$$

where $\theta_{i}$ represents individual *i*’s parameter estimate, θ represents the typical parameter estimate in the population, and $\eta_{i,\theta }$ represents the interindividual variability for individual *i*, which is normally distributed with a zero mean and variance. $\omega^{2}$ Interoccasion variability between dose occasions was investigated on absorption parameters (equation 2).

(2) $$\theta_{\mathrm{ij}}=\theta \times e^{\eta_{i,\theta }+\kappa_{j,\theta }}$$

where $\kappa_{j,\theta }$ represents the interoccasion variability of the pharmacokinetic parameter θ at the *j*’th occasion. The residual unexplained variability was assumed to be additive on a logarithmic scale.

Body weight was implemented as an allometric function on all clearance (exponent of 0.75) and volume of distribution (exponent of 1) parameters. Additionally, the effect of the enzyme maturation process of clearance in young children was also applied (equation 3).

(3) $${\text{CL}}_{i}=\text{TVCL}\times \frac{{\text{AGE}}_{i}^{\text{Hill}}}{{\text{MF}}_{50}^{\text{Hill}}+{\text{AGE}}_{i}^{\text{Hill}}}\times e^{\eta_{i,\text{CL}}}$$

where CL*_i_* represents the individual clearance parameter, TVCL represents the typical population mean value of the elimination clearance, AGE*_i_* represents individual age, MF_50_ represents the age corresponding to 50% enzyme maturation, and Hill represents the slope of the maturation function. Physiologically relevant demographic covariates, including body temperature, malaria parasite count, hemoglobin, total bilirubin, AST, ALT, serum creatinine, BUN, potassium, and chloride, were investigated with a stepwise covariate approach. A stepwise forward inclusion and backward deletion approach (*P* values of <0.05 and <0.001 for forward and backward step, respectively) were implemented in Pearl-speaks-NONMEM (PsN; version 3.6.0).

### QT interval correction methods.

All QT intervals were corrected for heart rate before further analyses were conducted. Four different correction methods were used to calculate QTc intervals. The most commonly used formulas are the Fridericia correction (equation 4) and Bazett correction (equation 5). The QTc_F_ and QTc_B_ intervals were used as a reference in this study.

(4) $${\text{QTc}}_{\text{F}}=\frac{\text{QT}}{\sqrt[{3}]{\text{RR}}}=\text{QT}\times {\text{RR}}^{-0.33}$$

(5) $${\text{QTc}}_{\text{B}}=\frac{\text{QT}}{\sqrt{\text{RR}}}=\text{QT}\times {\text{RR}}^{-0.5}$$

where QT represents the measured QT interval, in milliseconds, and RR represents the RR interval, in seconds. Study-specific correction factors were investigated using the observed data generated in this study. Pretreatment QT and RR measurements were transformed into their natural logarithms and used to estimate a study-specific correction factor by applying a simple linear regression analysis (equation 6).

(6) Ln(QT)=β+α×Ln(RR)

where β represents the intercept and α represents the slope of the regression. The estimated slope of the regression model was implemented as the correction factor and applied to all QT interval measurements in the study (equation 7):

(7) $$\text{QTc}=\text{QT}\times {\text{RR}}^{-\alpha }$$

Furthermore, QT interval measurements were also stratified on the day of measurement (i.e., day 1, day 3, and day 7), and a specific correction factor was estimated for each day and applied as a correction for data collected on that day (QTc_DAYS_). QT intervals, corrected by the four methods (QTc_F_, QTc_B_, QTc_SSB_, and QTc_DAYS_), were used to investigate the effect of piperaquine on QTc prolongation. The slopes of the linear regression between QTc and RR intervals were used to evaluate the performance of each correction method (21). A slope close to zero represents a complete correction of the QTc calculations across the heart rate range and a consistent and appropriate performance of the method.

### Clinical determinants of QTc prolongation.

According to ICH-E14, the guidance for the clinical evaluation of QT/QTc interval prolongation and proarrhythmic potential for non-antiarrhythmic drugs (44), the following categories of QTc interval prolongations should be used as reference limits for clinical analysis: absolute QTc interval, ≤450 ms, 451 to 480 ms, >481 to 500 ms, and >500 ms; ΔQTc interval, ≤30 ms, 31 to 60 ms, and >60 ms. These categories were used as a reference in this study for the categorical analysis. Factors associated with the risk of QTc interval prolongation were evaluated in each of the above categories of QTc interval prolongation. The list of the non-drug factors associated with QTc interval prolongation (moderate to high quality of evidence) and the list of drugs that prolong QTc interval stated at www.Crediblemeds.org as known, possible, and conditional risks of TdP were used as references (45). Non-drug factors associated with QTc prolongation included in the analysis were female gender, age, potassium level, hypokalaemia (<3.5 mmol/liter), body temperature, and QTc interval at enrollment. Potential differences between groups were compared with Kruskal-Wallis test or Dunn’s test for continuous variables and chi-square test or Fisher’s exact test for categorical variables.

### Relationship between piperaquine concentration and QTc interval.

Individually predicted piperaquine concentration-time profiles were constructed based on the empirical Bayes (*post hoc*) estimates (EBEs) from the final population pharmacokinetic model. The relationship between piperaquine concentrations and the absolute QTc interval was evaluated using the best-performing QT interval correction method from the previous stage. The linear and *E*_max_ functions were evaluated as shown in equations 8 and 9, respectively.

(8) $$\text{QTc}\left(t\right)=({\text{QTc}}_{\text{Baseline}}+\eta )+\left(\text{slope}\times \mathrm{Cp}\right)+\varepsilon_{i}$$

(9) $$\text{QTc}\left(t\right)=({\text{QTc}}_{\text{Baseline}}+\eta )+\left(E_{\text{max}}\times \frac{C_{p}^{\gamma }}{C_{p}^{\gamma }+{\text{EC}}_{50}^{\gamma }}\right)+\varepsilon_{i}$$

where QTc_Baseline_ represents the baseline value of the QTc interval (in milliseconds) at enrollment. Slope represents the slope of the linear relationship of piperaquine and QTc interval (QTc prolongation in milliseconds per 100 ng/ml), *C_p_* represents the piperaquine concentration (in nanograms per milliliter), *E*_max_ represents the maximum QTc interval (in milliseconds) achieved at infinite drug concentration, EC_50_ represents the piperaquine concentration (in nanograms per milliliter) generating half of the maximum drug effect, γ represents the Hill factor, η represents the interindividual variability, and $\varepsilon_{i}$ represents the residual error. The influence of patient characteristics on pharmacodynamic parameters was investigated using a stepwise covariate approach, as described for the pharmacokinetic model-building process.

### Model diagnostics and evaluation.

Model diagnostics and automation were performed using Xpose version 4.0, Pirana, and PsN. Goodness-of-fit and simulation-based diagnostics were used to evaluate the descriptive and predictive performances of the model. The robustness of parameter estimates from the final model was performed using 1,000 bootstrap runs. Numerical and visual predictive checks (*n* = 2,000) were used to evaluate the predictive performance of the final model. The individual parameter estimates from the final pharmacokinetic model were used further to generate the concentration-time profiles of piperaquine for the pharmacokinetic-pharmacodynamic analysis.

### Population-based simulations of clinical scenarios.

The updated dosing recommendation for dihydroartemisinin-piperaquine in the latest edition of the WHO *Guidelines for the Treatment of Malaria* (2015) suggested an increased dosage in young children (8). This adjustment was a strong recommendation based on pharmacokinetic modeling (9, 10). The final pharmacokinetic-pharmacodynamic model describing the effect of piperaquine on the absolute QTc interval was used to simulate the impact of the newly recommended dose as well as the previous recommendation. The impact on absolute QTc interval was assessed both in acute treatment and in mass drug administration settings.

Once-daily dosing of dihydroartemisinin-piperaquine for 3 days was simulated to evaluate the cardiac safety of the different dosing regimens in the acute malaria treatment scenario. For mass drug administration, a full 3-day treatment regimen of dihydroartemisinin-piperaquine, once a month for a total of 3 months, was simulated to evaluate the cardiac safety of the different dosing regimens. The detailed dosing regimens used are summarized in Table 5. The relationship between age and body weight in the study population was used to assign an age to each simulated patient at different body weights. A total of 480,000 patients were simulated, including 5,000 patients at each body weight (5 to 100 kg) for each dosing scenario.

## Supplementary Material

Supplemental file 1
