# Supplementary material for: Pooled Multicenter Analysis of Cardiovascular Safety and Population Pharmacokinetic Properties of Piperaquine in African Patients with Uncomplicated Falciparum Malaria
Source: Antimicrob Agents Chemother. 2020 Jun 23;64(7):e01848-19. doi: 10.1128/AAC.01848-19 (PMC7318010; doi:10.1128/AAC.01848-19)
Supplement: Supplemental file 1 [file AAC.01848-19-s0001.pdf]

Supplementary material

Relationship between piperaquine exposure and ΔQTc-interval

Additional analyses were conducted to investigate the relationship of piperaquine concentration and ΔQTc-interval, in order to compare with the values previously reported in the literature. The relationship between piperaquine concentrations and ΔQTc-intervals was assessed initially by using a linear model (Equation 1). The magnitude of the piperaquine effect on the ΔQTc-interval was compared between each correction method. Furthermore, the best performing model was used for further investigation, by implementing an E_{max} function to describe the drug effect instead of a linear function (Equation 2).

$$\Delta QTc = (\Delta QTc_{Baseline} + \eta) + (Slope + \eta) \times C_P + \varepsilon_i \qquad \text{(Eq. 1)}$$

$$\Delta QTc = (\Delta QTc_{Baseline} + \eta) + \left(E_{max} \times \frac{C_p^{\gamma}}{C_p^{\gamma} + EC_{50}^{\gamma}}\right) + \varepsilon_i \qquad \text{(Eq. 2)}$$

where $\Delta QTc_{Baseline}$ represents the baseline ΔQTc-interval (ms), Slope represents the slope of the relationship between piperaquine and ΔQTc-interval (QTc-interval ms prolongation per 100 ng/ml increase in piperaquine concentration), C_p represents the piperaquine concentration (ng/ml), E_{max} represents the maximum ΔQTc-interval (ms) achieved at infinite drug concentration, EC_{50} represents the piperaquine concentration (ng/ml) generating half of the maximum drug effect, γ represents the hill factor, η represents the inter-individual variability and ε_i represents the residual error. The influence of patient characteristics on pharmacodynamic parameters was investigated using a stepwise covariate approach as described in the pharmacokinetic model building process (main manuscript).

Results from the linear model, using QT-interval corrected by the study specific correction factor (QTc_{SSB}), demonstrated a significantly better model fit compared to all other correction approaches (ΔOFV = -961, $p < 0.001$, compared to a model using QTc_F). The four different correction methods resulted in large differences in the estimated relationship between piperaquine concentration and ΔQTc-interval (i.e. slope of the concentration-response model). A linear regression analysis of piperaquine concentrations *vs* ΔQTc_F, ΔQTc_B, ΔQTc_{SSB}, and ΔQTc_{DAYS} resulted in a predicted 7.97ms, 5.30ms, 5.90ms, and 4.11ms QTc-prolongation, respectively, per 100 ng/ml increase in piperaquine concentration (Table S1).

The model using QTc_{SSB} was investigated further by implementing an E_{max} function, which showed a substantially improved model fit compared to a linear model (ΔOFV = -525, ΔBIC = -500). A stepwise covariate search resulted in age as a significant covariate on the concentration needed for half of maximum effect (EC_{50}; ΔOFV = -15.8). The parameter estimates of the final pharmacokinetic-pharmacodynamic model describing the piperaquine effect on the ΔQTc-interval are summarized in Table S2. The goodness-of-fit diagnostics and visual predictive check are shown in Figure S4.

As in the categorical analysis, we found that patients with shorter baseline QTc-interval tended to have higher QTc-prolongation. Thus, we also investigated the effect of baseline QTc-interval on the slope of the linear relationship between QTc-prolongation and piperaquine exposure.

This covariate effect improved the model using QTcF as observations, where the slope decreased with the increase in baseline value. However, this covariate effect was not found to be significant in the models using QTc_B, QTc_{SSB}, and QTc_{DAYS}. This was supported further by the E_{max} model, in which the maximum QTc-prolongation (E_{max}) and EC_{50} were unaffected by the baseline QTc-interval. Thus, the baseline QTc-interval was not included as a covariate in the final model.

These results demonstrated that the correction method had a large impact on the magnitude of the predicted drug effect and that standard correction methods, such as Fridericia or Bazett, may not always be appropriate. We have shown here that, where possible, study specific correction should be evaluated and applied if the estimated correction performs better than the conventional correction methods, to minimize the impact of varying heart rates in electrocardiographic drug evaluations.

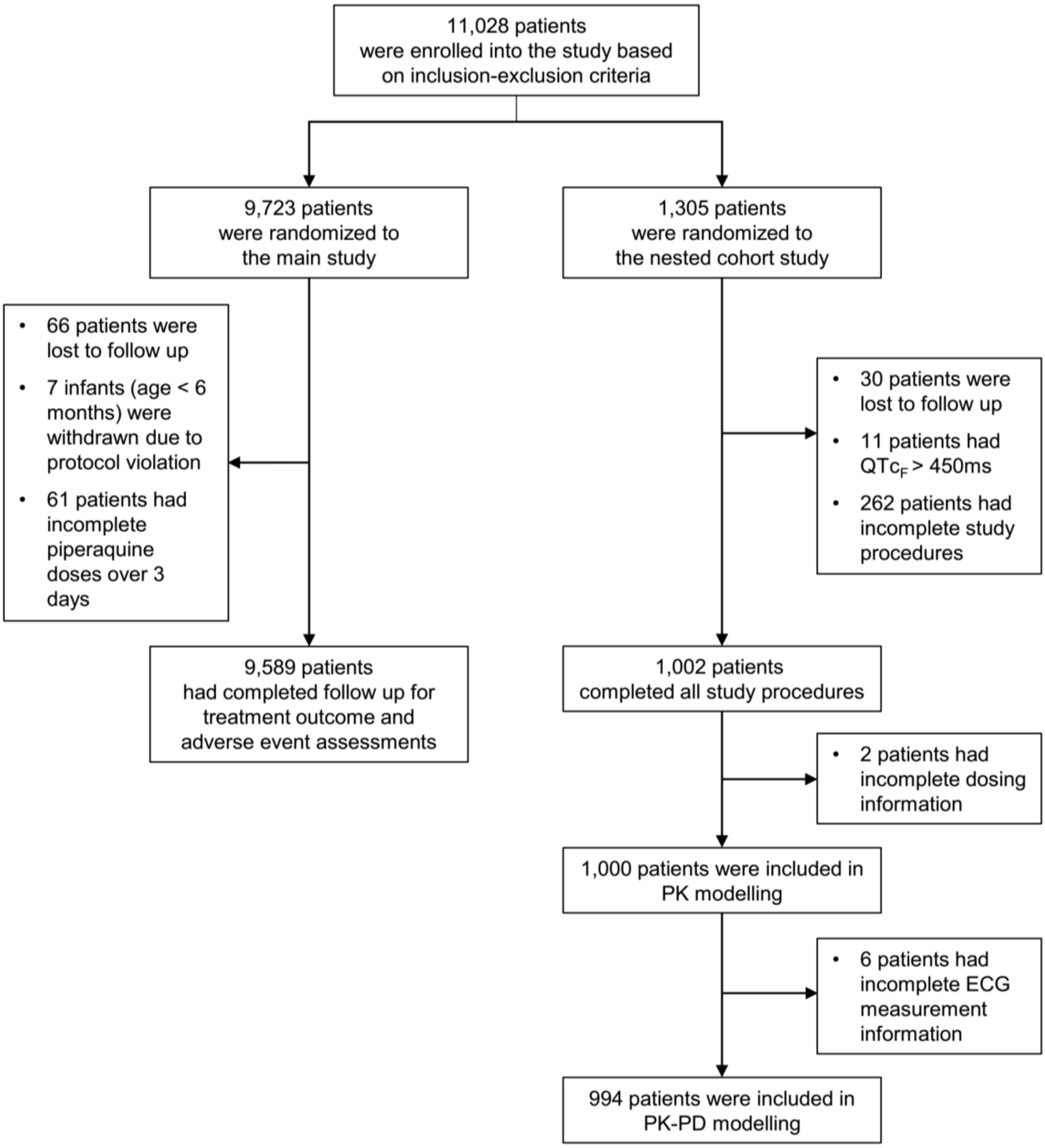

Figure S1. Study diagram.

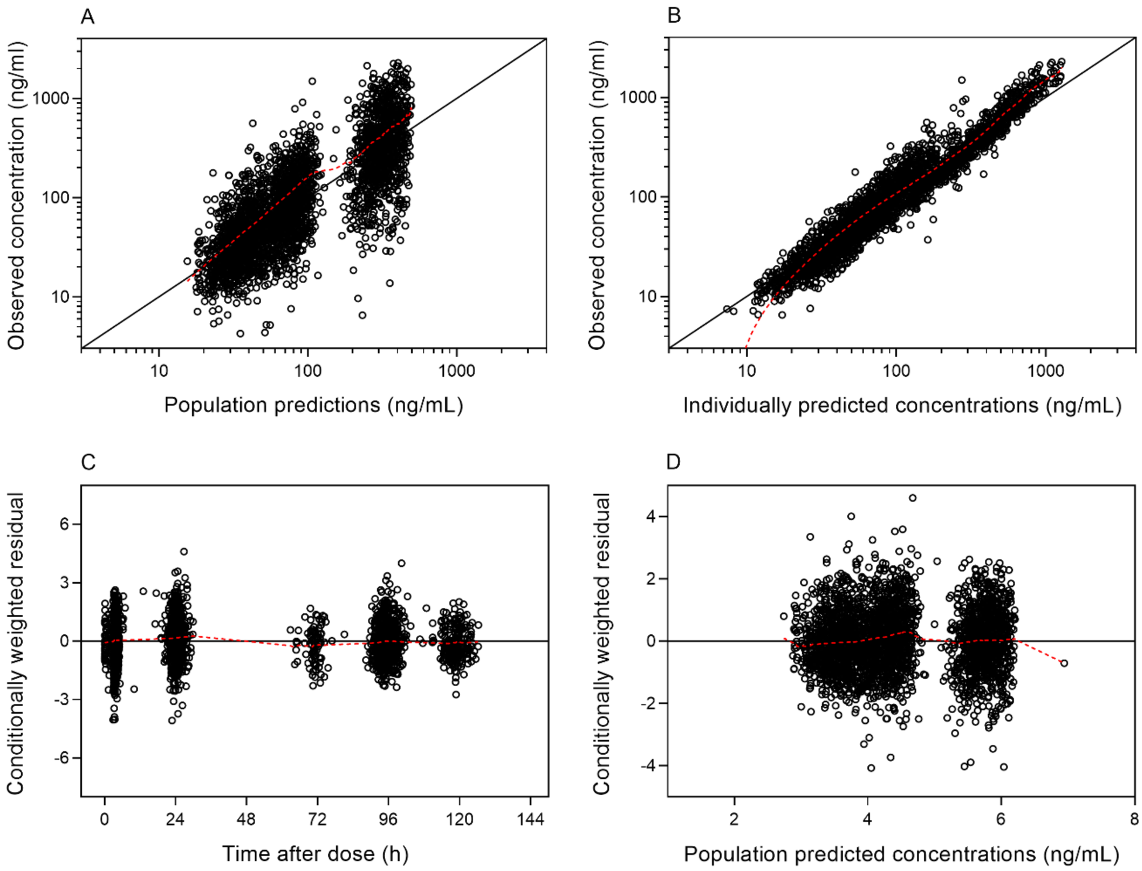

Figure S2. Diagnostics of the final piperaquine pharmacokinetic model.

Goodness-of-fit plots showing (A) observed concentrations *vs* population predictions, (B) observed concentrations *vs* individually predicted concentrations, (C) conditionally weighted residual *vs* time after dose, and (D) conditionally weighted residual *vs* population predictions. The open circles represent the observed piperaquine concentrations. The solid black lines represent the line of identity or zero-line and the dashed red lines represent a local polynomial regression fitting of all observations (i.e. trend line).

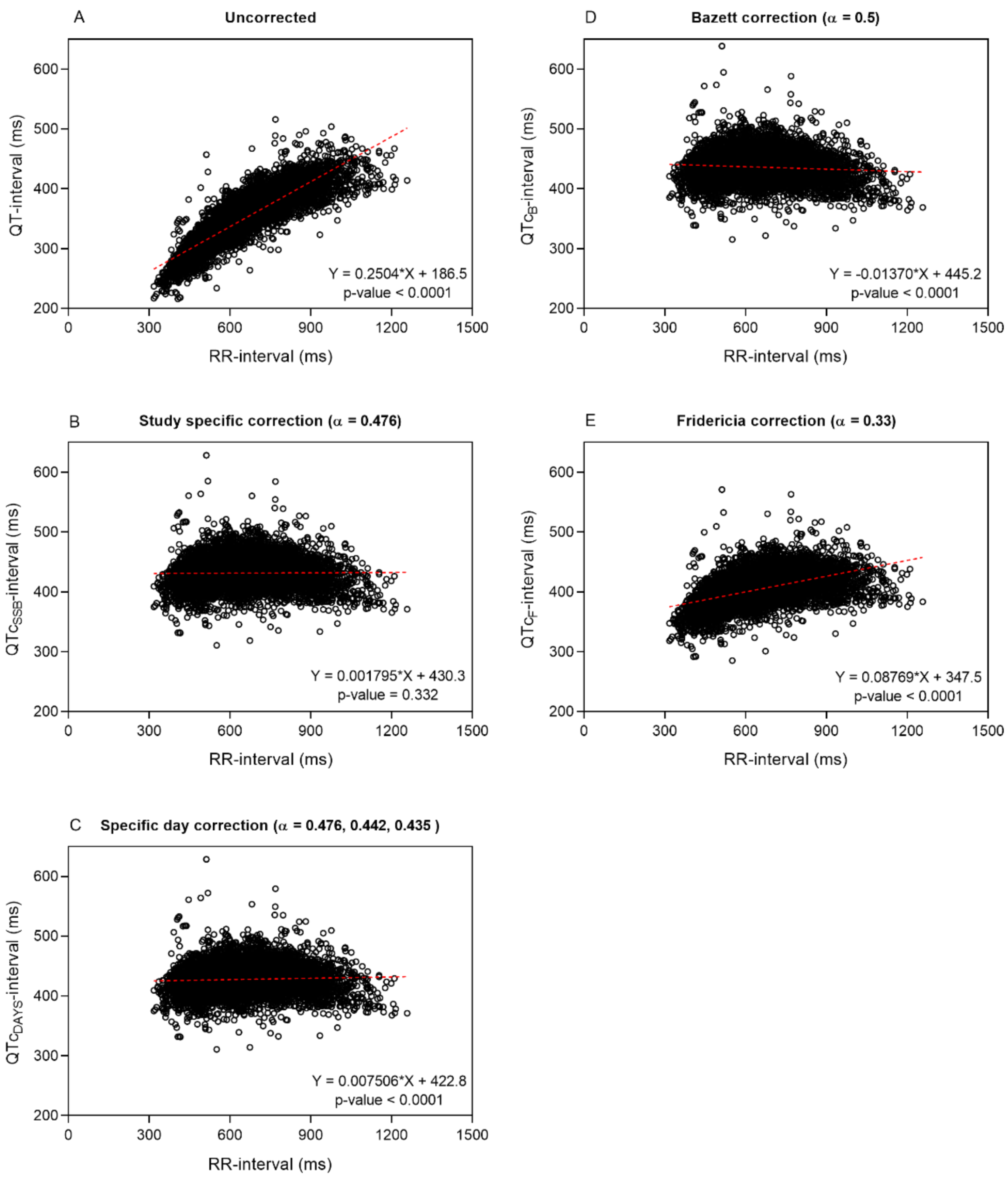

Figure S3. Relationship between QTc-interval and RR-interval, using different heart rate correction methods.

All correction methods used the following general equation to correct the measured QT-interval for heart rate: $QTc = QT \times RR^{-\alpha}$. (A) Uncorrected QT-interval, (B) QTc-interval corrected by a study specific correction factor (QTc_{SSB}, α = 0.476), (C) QTc-interval corrected by a specific day correction factor (QTc_{DAYS}, α = 0.476, 0.442, 0.435), (D) QTc-interval corrected by Bazett correction (QTc_{B}, α = 0.5), and (E) QTc-interval corrected by Fridericia correction (QTc_{F}, α = 0.33). The linear regression (dashed red line) and associated equations of each correction method are presented within each figure.

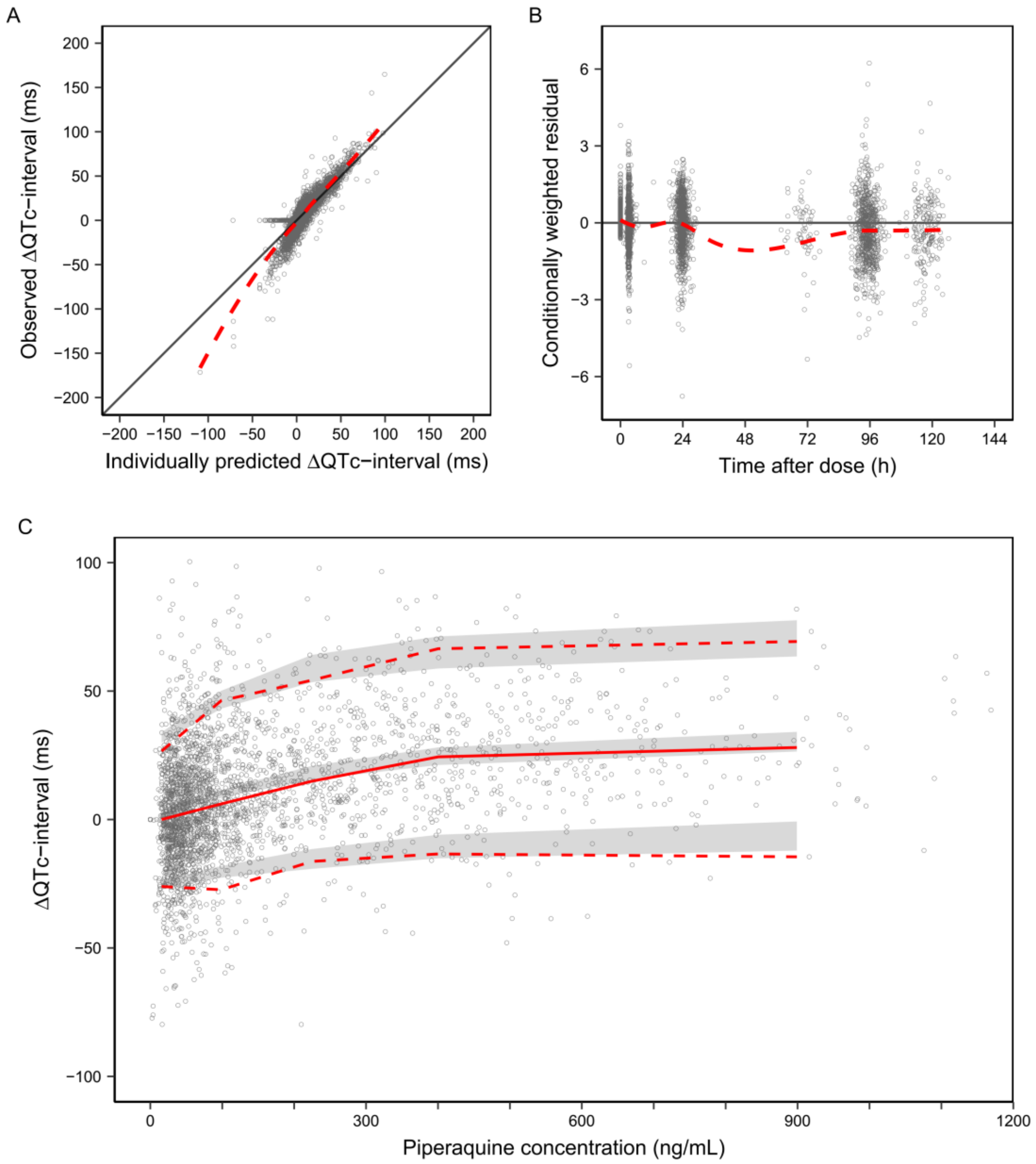

Figure S4. Diagnostics of the final model describing relationship between piperaquine concentrations and ΔQTc-interval using an E_{max} function.

Goodness-of-fit plots showing (A) observed ΔQTc-interval *vs* individually predicted ΔQTc-interval, and (B) conditionally weighted residual *vs* time after dose. The solid black lines represent the line of identity and the dashed red lines represent a local polynomial regression fitting of all observations (i.e. trend line). Visual predictive check (C) of the final model, describing the relationship between piperaquine concentrations and ΔQTc-interval using an E_{max} function (n=2,000). The open circles represent the observed data. Solid red lines represent the 50th percentile of the observations, and dashed red lines represent the 5th and 95th percentiles of the observations. The shaded areas represent the 95% confidence intervals of each simulated percentile.

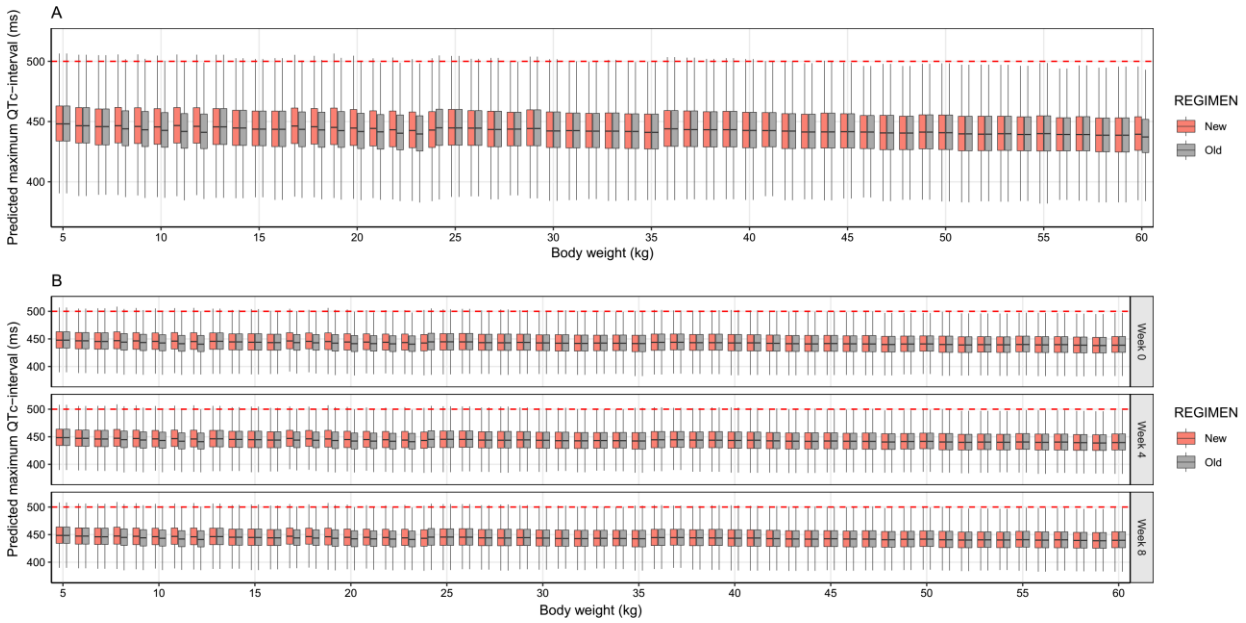

Figure S5. Predicted maximum QTc-intervals after different dosing regimens, simulated from the final pharmacokinetic-pharmacodynamic model.

The box plots represent the simulated maximum QTc-interval, stratified by body weight after receiving the old and new dosing regimen for (A) acute malaria treatment (3-day regimen) and (B) mass drug administration (3-day monthly regimen). The dashed red lines represent an absolute QTc-interval regulatory safety cut-off of 500 ms.

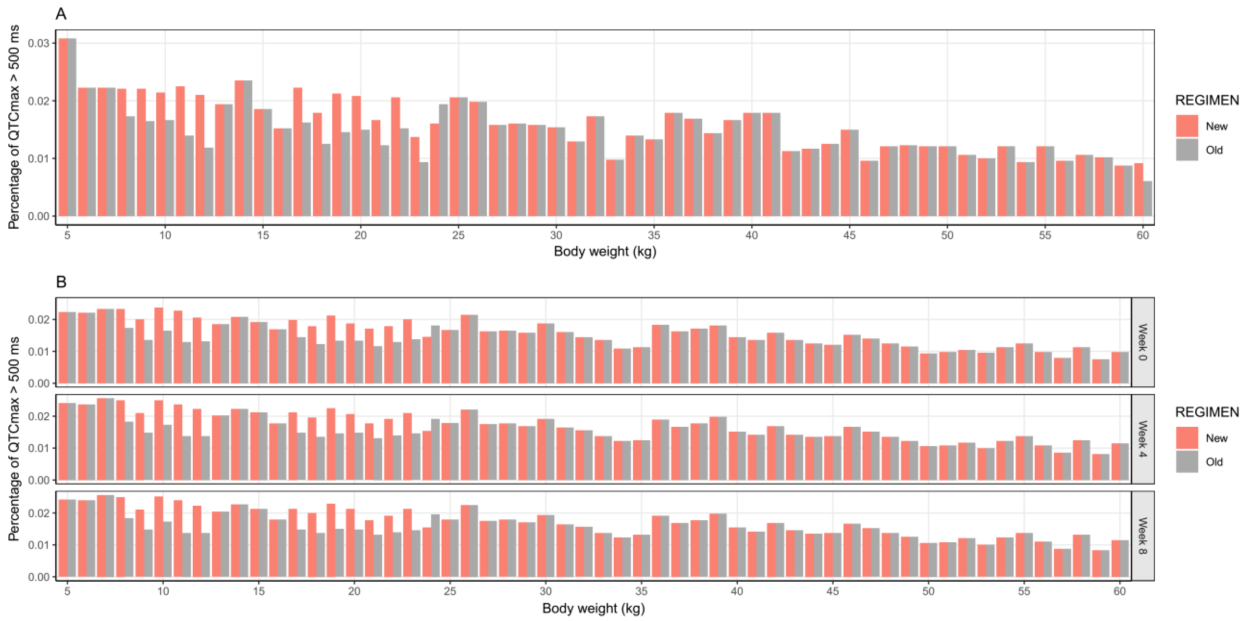

Figure S6. Predicted probability (risk) of having a maximum QTc-interval of >500 ms after different dosing regimens, simulated from the final pharmacokinetic-pharmacodynamic model.

The bar chart represents the probability (risk) of having a maximum QTc-interval of >500 ms based on a total of 480,000 simulated patients (5,000 simulated individuals per body weight, 5 to 100 kg) after receiving the old (grey bars) and new (red bars) dosing regimen, for (A) acute malaria treatment (3-day regimen) and (B) mass drug administration (monthly 3-day regimen).

Table S1. Relationship between ΔQTc-interval and piperaquine concentration, utilizing different heart rate correction methods.

Parameter	QT-interval correction method			
	QTc_F α = 0.333 (%RSE)[a]	QTc_B α = 0.500 (%RSE)[a]	QTc_{SSB} α = 0.476 (%RSE)[a]	QTc_{DAYS} α = 0.476, 0.442, 0.435 (%RSE)[a]
OFV	27,224	26,530	26,263	26,340
ΔOFV	-	-694	-961	-884
Baseline	0 fixed	0 fixed	0 fixed	0 fixed
IIV Baseline	15.5 (12.8)	14.0 (18.3)	13.7 (18.3)	14.0 (17.7)
Slope (ms/100 ng/ml)	7.97 (4.39)	5.30 (5.09)	5.90 (4.07)	4.11 (4.38)
IIV Slope	0.253 (20.0)	0.122 (30.2)	0.128 (25.3)	0.076 (37.5)
σ (ms)	15.5 (5.64)	14.8 (6.10)	14.1 (6.23)	14.6 (5.90)

Abbreviations: OFV, objective function value; ΔOFV, the difference in OFV compared to the model using Fridericia correction; σ, additive residual error of QTc-interval measurements; α, correction factor; IIV, additive inter-individual variability.

[a] Computed population mean parameter estimates from NONMEM. IIV are presented as absolute variability on an arithmetic scale. Parameter precision is presented as relative standard deviation (%RSE), calculated as $100\times\frac{\text{Standard error}}{\text{Final parameter estimate}}$.

Table S2. Parameter estimates from the final pharmacokinetic-pharmacodynamic model for the piperaquine effect on ΔQTc-interval.

Parameter	Population estimate[a] (% RSE)[b]	95% CI[b]	IIV %CV (% RSE)[b]	95% CI[b]
Piperaquine effect on ΔQTc-interval				
$\Delta QTc_{Baseline}$	0 fixed	-	10.6[c] (15.2)	8.05-14.1
E_{max} (ms)	47.5 (9.48)	42.1-53.4	29.6[d] (22.9)	15.8-43.5
EC_{50} (ng/ml)	319 (16.8)	260-409	217 [d] (7.82)	150-290
γ	1.22 (6.41)	1.11-1.35	-	-
Effect of age on EC_{50} (%)	2.87 (35.2)	1.31-4.80	-	-
σ (ms)	12.6 (3.72)	11.7-13.5	-	-

Abbreviations: $\Delta QTc_{Baseline}$, change of QTc-interval compared to baseline; E_{max}, maximum ΔQTc-interval associated with drug effect; EC_{50}, piperaquine concentration needed to achieve 50% of the maximum drug effect; γ, shape function of the E_{max} model; σ, additive residual error (variance) of ΔQTc-interval measurements; IIV, inter-individual variability.

[a] Computed population mean parameter estimates from NONMEM.

[b] Based on nonparametric bootstrap diagnostics (n = 1,000). Parameter precision is presented as relative standard deviation (%RSE), calculated as $100\times\frac{\text{Standard deviation}}{\text{Mean value}}$.

[c] Additive inter-individual variability, presented as absolute variability on an arithmetic scale.

[d] Exponential inter-individual variability, presented as the coefficient of variation (%CV), calculated as $100 \times \sqrt{\text{exp(estimate)-1}}$.

Table S3. Number and percentage of patients with absolute QTc-interval and ΔQTc-interval at different thresholds according to ICH-E14, the guidance for clinical evaluation of QT/QTc-prolongation.

Threshold	Observed QTc_F (n=994)	Observed QTc_B (n=994)	Observed QTc_{day1} (n=994)	Simulations from final model using QTc_{day1} (n=994,000)	Simulated data (n=960,000)	
					Old regimen (n=480,000)	New regimen (n=480,000)
ΔQTc-interval						
≤30ms	490 (49.3%)	668 (67.2%)	638 (64.2%)	654,435[a] (65.8)	376,781 (78.5%)	365,822 (76.2%)
31-60ms	368 (37.0%)	257 (25.9%)	286 (28.8%)	296,315[a] (29.8%)	93,726 (19.5%)	103,366 (21.5%)
>60ms	136 (13.7%)	69 (6.94%)	70 (7.0%)	43,250 [a] (4.35%)	9,493 (1.98%)	10,812 (2.25%)
QTc-interval						
≤450ms	852 (85.7%)	399 (40.1%)	481 (48.4%)	635,681[b] (64.0%)	298,487 (62.2%)	286,469 (59.7%)
451-480ms	124 (12.5%)	459 (46.2%)	408 (41.0%)	288,962[b] (29.1%)	153,288 (31.9%)	162,063 (33.8%)
481-500ms	12 (1.21%)	100 (10.1%)	78 (7.8%)	50,634[b] (5.09%)	22,976 (4.79%)	25,551 (5.32%)
>500ms	6 (0.60%)	36 (3.62%)	27 (2.7%)	18,723[b] (1.88%)	5,249 (1.09%)	5,917 (1.23%)

Abbreviations: ΔQTc-interval, QTc-prolongation from baseline measurement; QTc-interval, the absolute QTc-interval; QTc_F, QTc-interval using Fridericia correction; QTc_B, QTc-interval using Bazett correction, and QTc_{SSB}, QTc-interval using pre-treatment correction. The numbers of patients having observed QTc_F, QTc_B, and QTc_{SSB} in each category were based on observations measured after the treatment. The simulations were based on final model describing piperaquine effect on absolute QTc-interval.

[a] Simulated from the final model describing piperaquine effect on ΔQTc-interval.

[b] Simulated from the final model describing piperaquine effect on absolute QTc-interval.
